# Combined In Vitro and In Vivo Approaches to Propose a Putative Adverse Outcome Pathway for Acute Lung Inflammation Induced by Nanoparticles: A Study on Carbon Dots

**DOI:** 10.3390/nano11010180

**Published:** 2021-01-13

**Authors:** Maud Weiss, Jiahui Fan, Mickaël Claudel, Luc Lebeau, Françoise Pons, Carole Ronzani

**Affiliations:** Laboratoire de Conception et Application de Molécules Bioactives, Faculté de Pharmacie, UMR 7199, CNRS-Université de Strasbourg, 67400 Illkirch, France; maudweiss@unistra.fr (M.W.); jiahui.fan@etu.unistra.fr (J.F.); mickael.claudel@etu.unistra.fr (M.C.); llebeau@unistra.fr (L.L.); pons@unistra.fr (F.P.)

**Keywords:** adverse outcome pathway, nanoparticles, carbon dots, nanotoxicology, lung toxicity, lysosome

## Abstract

With the growth of nanotechnologies, concerns raised regarding the potential adverse effects of nanoparticles (NPs), especially on the respiratory tract. Adverse outcome pathways (AOP) have become recently the subject of intensive studies in order to get a better understanding of the mechanisms of NP toxicity, and hence hopefully predict the health risks associated with NP exposure. Herein, we propose a putative AOP for the lung toxicity of NPs using emerging nanomaterials called carbon dots (CDs), and in vivo and in vitro experimental approaches. We first investigated the effect of a single administration of CDs on mouse airways. We showed that CDs induce an acute lung inflammation and identified airway macrophages as target cells of CDs. Then, we studied the cellular responses induced by CDs in an in vitro model of macrophages. We observed that CDs are internalized by these cells (molecular initial event) and induce a series of key events, including loss of lysosomal integrity and mitochondrial disruption (organelle responses), as well as oxidative stress, inflammasome activation, inflammatory cytokine upregulation and macrophage death (cellular responses). All these effects triggering lung inflammation as tissular response may lead to acute lung injury.

## 1. Introduction

Since the beginning of the 21st century, engineered nanoparticles (NPs) have been developed for a wide variety of industrial and biomedical applications [1]. Among these NPs, carbon dots (CDs) are the most recent member of the carbon nanomaterial family to be discovered [2]. CDs are spherical and very small-sized (2–50 nm) nanomaterials that exhibit water solubility, intrinsic fluorescence properties and chemical- and photo-stability [3]. They can be easily synthesized by top-down or bottom-up approaches. In the top-down method, CDs are produced by breaking down large carbon structures, such as graphite, graphene or carbon nanotubes. In the most popular bottom-up method, they are synthesized using various processes by the carbonization of organic matter (citric acid, glucose, fruit juice, etc.) in the presence of catalysts and/or passivation agents [4]. By varying the synthesis methodology and the nature of the carbon source, catalyst and passivating agent, the intrinsic characteristics of CDs can be modulated to give them specific physicochemical and optical properties [5,6]. Due to their unique properties, CDs have great potential for applications in various fields, including electronics, catalysis, solar technology or photovoltaics [7]. They are also promising candidate for applications in nanomedicine [8]. Thanks to their intrinsic photoluminescence properties and resistance to photobleaching, CDs are especially valuable for optical imaging and stand as a favorable alternative to heavy metal-based quantum dots and organic dyes [9]. Besides, CDs have gradually emerged as novel nano-carriers for drugs such as paclitaxel or doxorubicin [10,11]. Our group and others are also investigating CDs as gene delivery systems [5,12,13]. Thanks to the combination of imaging and drug delivery approaches, CDs are increasingly gaining ground as powerful candidates for the development of theranostic applications [14].

Because of their specific properties at the nanoscale, NPs may cause toxic effects at the cell and tissue levels [15]. This is particularly true in the lung, which represents the main route of entry of NPs in the body [16]. Due to their small size, inhaled NPs deposit all throughout the airways, from the bronchi to the alveoli, where they particularly accumulate when compared to larger particles [17]. In alveolar spaces, NPs can be internalized by different cell types, including epithelial cells and macrophages, as it has been reported in mice exposed to carbon nanotubes [18]. If macrophages play a central role in the removal of NPs, their activation after particle uptake may lead to the release of pro-inflammatory cytokines (TNF-α, interleukine (IL)-1β, IL-6 or IL-8) responsible for an influx of other immune cells such as neutrophils [19]. Besides, activated phagocytes may be an important source of reactive oxygen species (ROS) [20]. As a consequence, several kinds of engineered NPs, such as carbon black, silica or TiO_2_ NPs or carbon nanotubes, have been reported to induce acute lung inflammation in animal models [21]. Hence, developing engineered NPs for industrial or biomedical applications requires assessing their safety, especially in the lung.

A key role of nanotoxicology is to understand NP toxicity mechanisms in order to predict the associated health risks [22]. In response to the challenge, and as it is not possible to experimentally test each NP, the identification of adverse outcome pathways (AOPs) has been recently proposed [23]. An AOP is a framework which gathers the existing knowledge on the links between molecular initiating events (MIEs) and adverse outcomes (AOs) induced by a xenobiotic by describing the sequential chain of measurable key events (KEs) at different levels of biological organization that are required to produce a toxic response [24]. To date, more than 200 AOPs are under development, mainly on chemical compounds, and less than about fifty AOPs have been endorsed by the Organization for Economic Cooperation and Development (OECD) [25]. In the lung toxicology field, the AOP for lung fibrosis in response to chemicals (AOP 173) is currently the most advanced (https://aopwiki.org/aops/173), and it is suggested that this AOP would be applicable to NPs [26]. Indeed, some studies conducted on carbon nanotubes showed that mechanisms of carbon nanotube-induced lung fibrosis are common to those described in AOP 173 [27,28]. Other AOPs being currently developed in the field of inhalation toxicology, such as AOP for acute lung toxicity, lung emphysema or lung cancer, are potentially relevant for nanomaterials. But, contrary to chemicals whose MIEs are often well defined (ligand-receptor binding or protein modification), the MIEs and KEs involved in the toxicity cascade evoked by NPs are not yet fully understood [23]. Thus, developing more AOPs specifically related to lung toxicity induced by NPs is essential.

In this context, we combined in vivo and in vitro experimental approaches to propose an AOP for the lung toxicity of CDs. CDs are often described as biocompatible, but some studies recently pointed out a possible toxicity of these nanomaterials [29,30,31]. In fact, the extreme diversity of the physicochemical characteristics of CDs complicates their safety evaluation. Thus, our team recently explored the impact of the physicochemical characteristics of CDs on their toxicity using a library of 35 CDs with various chemical composition, size, charge and surface chemistry [32]. Among several factors, our data highlighted that surface chemistry and charge strongly impact the toxicity of CDs. In particular, polyamine surface-passivated cationic CDs, which are one type of CDs developed for gene delivery applications, have been shown to be cytotoxic to macrophages. In line with our data, poly(ethyleneimine) (PEI)-based CDs were reported to be cytotoxic towards fibroblasts [29], or kidney and liver cells [30]. Furthermore, CDs passivated with ethylenediamine caused injury and inflammation in the lung of mice after a single inhalation exposure [31]. It is therefore important to understand how these NPs exert their toxicity, and how it is possible to overcome it, in order to fully exploit their therapeutic potential. Indeed, the use of CDs as nano-carriers remains a great opportunity, in particular in the fields of theranostic and gene delivery, as CDs are less toxic than, e.g., the standard delivery reagent used for DNA transfection, namely bPEI25k [13]. Production of ROS [29,33], inflammation [34] or activation of the autophagy process [33,35] have been proposed as CD toxicity mechanisms. Recently, our group also reported on the implication of the lysosome in the cytotoxicity of PEI-based CDs [36]. However, to our knowledge, integration of information about CD-induced toxicological events at the cellular and organ levels has not been investigated in the literature yet. In the present study, we first investigated the toxicological effects induced by polyamine-based cationic CDs in the lung of mice. We showed that these CDs induce lung inflammation and identify airway macrophages as their cell target in the organ. Then, we studied the cellular responses induced by these CDs in an in vitro model of macrophages. We thus observed that CDs are internalized by these cells (MIE) and induce loss of lysosomal integrity, mitochondrial disruption, oxidative stress, and inflammasome activation (KEs) leading to macrophage death and lung inflammation. We thus propose a putative AOP for acute lung injury induced by polyamine-based cationic CDs.

## 2. Materials and Methods

### 2.1. Preparation and Characterization of CDs

The CDs investigated herein were produced by microwave-assisted pyrolysis of organic precursors as reported previously [5,32]. In brief, 125 mg citric acid (Merck, Darmstadt, Germany), 500 mg branched poly(ethyleneimine) with molecular weight 600 Da (bPEI600, Sigma-Aldrich, St. Louis, MO, USA) and 5 mL HCl 0.1 N were mixed in an Erlenmeyer flask, and heated in a domestic microwave oven at a power regime of 620 W, for 170 s. The residue was then dissolved in HCl 0.1 N and centrifuged (10,000 rpm, 5 min). The resulting supernatant was loaded in a dialysis bag (MWCO 3500 Da) for dialysis against HCl 0.1 N (24 h) and ultra-pure water (24 h). The dialyzed preparation was lyophilized to yield a brown hygroscopic powder (275 mg).

The hydrodynamic diameter and size distribution of CDs, expressed as polydispersity index (PdI), were measured by dynamic light scattering (DLS, Zetasizer Nano ZS, Malvern Instruments, Paris, France) and calculated from the number distribution graph. Zeta potential was measured by DLS as well and calculated with the Smoluchowski’s equation. All measurements were performed in triplicate on fresh samples (1.0 mg/mL in 1.5 mM NaCl pH 7.4) at 25 °C. The morphology of CDs was observed by transmission electron microscopy (TEM) using a bench top LVEM5 microscope (Delong Instruments, Brno, Czech Republic) operating at 5 kV. CD samples (0.5 µL, 1.0 mg/mL) were deposited on glow-discharged (90 V and 2 mA for 15 s) carbon-coated grids (Cu-300HD, Pacific Grid Tech, San Francisco, CA, USA). After at least 2 h drying at room temperature, the grids were observed. The average size of the NPs was determined by image analysis using the ImageJ software (v 1.50i, NIH, Bethesda, MD, USA), from a set of 687 particles. Elemental composition of CDs was determined by analyzing carbon, hydrogen and nitrogen content (expressed as mass ratio) on a Vario EL III instrument (Elementar, Langenselbold, Germany). Other elements, including oxygen and chlorine, were not quantified. UV-visible and fluorescence measurements were recorded on CD samples prepared in ultra-pure water (0.10 mg/mL), with an UviKon XL spectrometer (Bio-Tek Instruments, VT, USA) and a Fluoromax-4 spectrofluorometer (Horiba Scientific, Kyoto, Japan), respectively, using a 1-mL quartz cuvette.

### 2.2. Animal Experimentation

Nine-week-old male BALB/c mice were purchased from Charles River Laboratories (Saint-Germain-sur-l’Arbresle, France). Animals were maintained under controlled environmental conditions with a 12-h/12-h light/dark cycle according to the EU guide for use of laboratory animals. Food and tap water were available ad libitum. The animals were acclimated for 1 week before the initiation of the study. Animal experiments were conducted in compliance with the European legislation (Directive 2010/63/EU). Experimental protocols were approved by the local ethics committee (CREMEAS, agreement number #4674). Mice were exposed to a single dose of 25, 50 or 100 μg CDs and CD-induced responses were measured 4 h, 24 h, 7 d, 14 d or 21 d later. CD samples (25 µL prepared in saline) were administered into the lung of animals by intranasal instillation. Administration was carried out under anesthesia with 50 mg/kg ketamine (Imalgen^®^, Merial, France) and 3.33 mg/kg xylazine (Rompun^®^, Bayer, France) given i.p. Doses of CDs were set to 25, 50 and 100 μg according to our previous studies on carbon nanotubes [18,37]. Control animals received instillation of the same volume of saline alone. The experiment was terminated by i.p. injection of a lethal dose of pentobarbital (100–150 mg/kg).

### 2.3. Lung Inflammation Measurement

Lung inflammation was assessed on bronchoalveolar lavage fluids (BALFs) and lung tissues from n = 6 mice. The trachea was cannulated to perform bronchoalveolar lavages. Lungs were lavaged by 6 instillations of 0.5 mL ice-cold saline supplemented with 2.6 mM ethylendiaminetetraacetic acid (EDTA). BALFs recovered from the two first instillations were centrifuged (200 g for 5 min at 4 °C), and the resulting supernatant was stored at −20 °C until cytokine measurements. Cell pellets recovered from the 6 instillations were resuspended in saline-EDTA and used to determine total and differential cell numbers. Total cell counts were determined using a Neubauer’s chamber. Differential cell counts were assessed on cytologic preparations obtained by cytocentrifugation (Cytospin 4, Thermo Scientific, Illkirch-Graffenstaden, France). Slides were stained with Microscopy Hemacolor^®^ (Merck, Darmstadt, Germany) and at least 400 cells were counted on each preparation. Macrophage, neutrophil, eosinophil and lymphocyte numbers were expressed as absolute numbers from total cell counts. Moreover, macrophages containing CDs were observed by optical microscopy. After BALF collection, lungs were perfused with ice-cold phosphate buffer solution (PBS) and fixed in 4% paraformaldehyde for histology. Fixed lungs were rinsed with PBS, dehydrated and embedded in paraffin using standard procedures. Tissue sections (5 µm) were then prepared and stained with hematoxylin and eosin (H&E) to observe inflammation in lung tissue.

### 2.4. Flow Cytometry Analysis of Cells Recovered in BALFs

Flow cytometry analysis of cells recovered in BALFs was conducted in n = 3 mice, according to procedures previously described in the literature [38,39]. After tracheotomy, lungs of mice were lavaged with 5 mL of PBS-EDTA, the recovered BALFs were centrifuged (5 min, 300 g), and the pelleted cells were resuspended in 500 µL of PBS-EDTA. Then, BALF cells were stained for 30 min with the following antibodies (all from BioLegend, San Diego, CA, USA): Alexa Fluor700 anti-mouse CD45 (clone 30-F11; 0.27 µg/mL) to identify leukocytes, and PE anti-mouse GR-1 (clone RB6-8C5; 0.06 µg/mL) and PE-Cy7 anti-mouse CD11c (clone N418; 0.24 µg/mL) to distinguish macrophages from neutrophils. To prevent non-specific binding of antibodies to Fc receptor, a blocking solution (TruStain FcX™, BioLegend, San Diego, CA, USA) was added to the antibody mix. Besides, to assess cell viability, cells were stained with the cell death marker TO-PRO^®^-3 (Invitrogen^TM^, Thermo Scientific, Illkirch-Graffenstaden, France) at 0.08 µM just before the flow cytometry analysis. Living cells were discriminated from dead cells according to their fluorescence intensity collected using an APC (red laser) channel. Cellular mortality was expressed as percent of positive cells. To investigate CD uptake by BALF cells, cell fluorescence was measured using the BV421 (violet laser) channel. CD-positive cells were discriminated from CD-negative cells by intensity of the collected fluorescence. Internalization results were expressed as percent of positive cells. Fluorescence measurements were carried out on a LSRFortessa X 20^TM^ flow cytometer (BD Biosciences, Le Pont de Claix, France) and collected data were analyzed using FlowJo^TM^ software (v 10.2, Ashland, OR, USA).

### 2.5. Cell Culture

THP-1 (TIB-202™, ATCC) cells were grown in culture flasks at 37 °C in a 5% CO_2_ humidified chamber using RPMI-1640 culture medium containing 2 mM L-glutamine, 0.05 mM 2-mercaptoethanol, 100 UI/mL penicillin, 100 μg/mL streptomycin, and 10% heat inactivated fetal bovine serum (all reagents from GIBCO, Thermo Scientific, Illkirch-Graffenstaden, France). For experiments, cells were seeded in appropriate culture devices and differentiated into macrophages overnight by adding 10 ng/mL phorbol 12-myristate 13-acetate (PMA, Sigma-Aldrich, St. Louis, MO, USA) to the culture medium.

### 2.6. Assessment of CD Cell Uptake and Internalization Mechanisms by Cultured Cells

Confocal laser scanning microscopy (CLSM) and fluorescence activated cell sorting (FACS) were used to assess CD uptake by cultured cells. For CLSM experiments, cells were seeded into 8-well IbiTreat μ-Slides (1.5 polymer coverslip, IBIDI^®^, Ibidi GmbH, Gräfelfing, Germany) at a density of 10^5^ cells/well, differentiated into macrophages, and incubated with CDs for 4 h. At the end of the incubation time, the cells were carefully washed with culture medium and the DSQ12S fluorescent probe (10 nM in PBS) [40] was added to the samples for 5 min to label the cell membrane. Then, the intracellular localization of CDs was observed using a Leica SP2 microscope equipped with a 63× oil immersion objective (NA = 1.2). CDs and the DSQ12S membrane probe were excited with 405 and 635 nm laser sources, respectively. For FACS, cells were seeded into 24-well plates at a density of 5 × 10^5^ cells/well, differentiated into macrophages, and incubated with CDs for 4 h. After CD exposure, the supernatant was discarded and cells were rinsed twice with PBS and harvested by trypsin treatment. Cell suspensions were then analyzed in the LSRFortessa X 20^TM^ flow cytometer and fluorescence of each sample (20,000 events) was collected using a BV421 (violet laser) channel. CD uptake was quantified by determining changes in the mean of the fluorescence intensity (MFI) of CD-treated cells compared to untreated cells. Results were expressed as the ratio of the MFI of CD-treated cells to the MFI of untreated cells. 

FACS was also used for identifying mechanisms of CD internalization by cultured cells. Energy dependent internalization was studied by pre-incubating cells at 4 °C for 30 min prior to addition of CDs. For studying implication of other internalization pathways, cells were pre-treated for 30 min with various pharmacological inhibitors: 25 μM cytochalasin D (micropinocytosis and phagocytosis), 25 µM chlorpromazine (clathrin-dependent pathway), 50 µM nystatin, 2.5 µM filipin III or 200 μM genistein (caveolae-dependent pathway), or 5 mM methyl-β-cyclodextrin (lipid rafts pathway). Inhibitors were selected according to the literature [41]. Absence of cytotoxicity of these inhibitors at the chosen concentrations was verified (data not shown). Results were expressed as percent of inhibition of CD uptake.

### 2.7. Cell Viability Assay

Cell viability was assessed by the methyl tetrazolium (MTT) assay. Cells were seeded into 96-well plates at a density of 10^5^ cells/well, differentiated into macrophages and incubated with increasing concentrations of CDs (3–200 μg/mL) for 24 h. The culture medium was then removed and stored at −80 °C until the cytokine assay, and cells were carefully washed with PBS before the addition of complete culture medium containing MTT (100 μL of a 1.0 mg/mL solution). After incubation for 1 h, culture medium was removed, and cells were lysed with DMSO. Absorbance of the resulting samples was read at 570 nm with a correction at 690 nm using a Multiskan FC reader (Thermo Scientific, Illkirch-Graffenstaden, France). Cell viability was expressed as the percentage of the absorbance of CD-treated cells relative to the absorbance of untreated cells.

### 2.8. Lysosomal Membrane Integrity Assay

Neutral red (NR) assay was used to assess lysosomal membrane integrity. Cells were seeded into 96-well culture plates at a density of 10^5^ cells/well, differentiated into macrophages, and incubated with increasing concentrations of CDs for 24 h. At the end of the incubation period, culture medium was removed and cells were carefully washed with PBS. Complete culture medium containing NR (200 μL of a 100 μg/mL solution) was added to the cells for dye incorporation into intact lysosomes. After a 3-h incubation period, culture medium was removed and cells were lysed with 1% acetic acid solution containing 50% ethanol to release the incorporated dye. Absorbance of the resulting samples was read at 570 nm with a correction at 690 nm. Results were expressed as the percentage of the absorbance of treated cells relative to the absorbance of non-exposed control cells.

### 2.9. Oxidative Stress Assessment

Oxidative stress was assessed by measuring changes in reduced glutathione (GSH) cellular levels using the 2,3-naphthalenedialdehyde (NDA) probe. Cells were seeded into 24-well culture plates at a density of 5 × 10^5^ cells/well, differentiated into macrophages and incubated for 4 h with increasing concentrations of CDs. At the end of the CD exposure, cells were washed with 5 mM EDTA, 40 mM NaH_2_PO_4_, 110 mM Na_2_HPO_4_ (pH 7.4), and lysed with 0.1% Triton X100^®^. Then, proteins were denatured and precipitated (0.1 M hydrochloric acid, 50% sulfosalicylic acid) before sample centrifugation (10,000 g, 15 min, 4 °C). Cell lysates were then incubated with the NDA probe for 25 min at 4 °C, before fluorescence measurement (*λ*_ex_ = 485 nm; *λ*_em_ = 528 nm) using a Varioskan multimode reader (Thermo Scientific, Illkirch-Graffenstaden, France). A calibration curve was used to calculate the amount of reduced GSH in the samples. This amount was then expressed in nmol of GSH per mg of protein. To do so, protein concentration in samples was determined using the bicinchoninic assay (BCA, Sigma-Aldrich, St. Louis, MO, USA) according to the manufacturer’s instructions.

### 2.10. Mitochondrial Membrane Potential Assay

Mitochondrial membrane potential was assayed using the JC-10 fluorescent probe (Sigma-Aldrich, St. Louis, MO, USA) according to the manufacturer’s instructions. Cells were seeded into 96-well culture plates at a density of 10^5^ cells/well, differentiated into macrophages, and incubated with increasing concentrations of CDs for 4 h. After the CD treatment, the cell culture supernatant was removed and the JC-10 probe (100 μL) was added to the cells for 1 h. Then, fluorescence of the samples was measured (functional mitochondria: *λ*_ex_ = 540 nm, *λ*_em_ = 590 nm; non-functional mitochondria: *λ*_ex_ = 490 nm, *λ*_em_ = 525 nm). The ratio of fluorescence intensity at 525 nm to fluorescence intensity at 590 nm was calculated for each sample. Data were expressed as the fold change of CD-exposed cells relative to the non-exposed control cells.

### 2.11. Cytokine Assay

IL-6, IL-8, IL-1β, KC and MCP-1 were measured in BALFs or in cell culture supernatants by ELISA according to the manufacturer’s instructions (R&D Systems, Lille, France).

### 2.12. Statistical Analysis

Experiments were carried out on n = 3–9 biological replicates (in vitro experiments) or on n = 3–6 mice (in vivo experiments). Data are presented as mean ± SEM. Statistical differences between groups were determined by Student *t*-test or analysis of variance (ANOVA) followed by Dunnett’s test, using the GraphPad Prism software (v 6.0, San Diego, CA, USA). Data were considered as significantly different when *p* < 0.05.

## 3. Results and Discussion

### 3.1. Characterization of CDs

**Figure 1:**
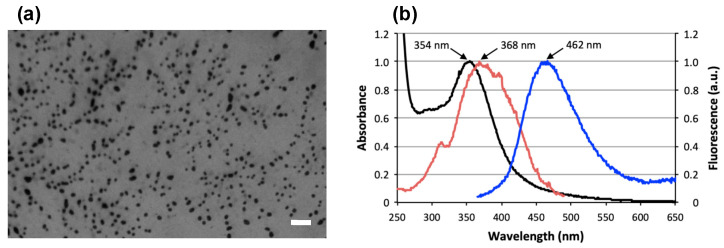
(**a**) Morphology of the bPEI600-based CDs as observed by TEM (scale bar = 200 nm). (**b**) Optical properties of the CDs: absorption (black), excitation (red), and emission (blue; excitation at 355 nm) spectra.

The mean hydrodynamic diameter of CDs was 10.1 ± 0.4 nm with a polydispersity index of 0.240 ± 0.061 (Table 1). The zeta potential (*ζ*) of the CDs was +23.9 ± 2.0 mV, indicating a positive surface charge of the NPs and revealing the presence of grafted poly(ethyleneimine) residues at the surface. CDs appeared as rounded particles on TEM images (Figure 1a), with a mean diameter of ca. 39.5 nm. The discrepancy between the size as determined by DLS and TEM might result from some aggregation of the NPs under capillary forces upon deposition and drying of the sample on the TEM grid. Nitrogen, carbon, and hydrogen contents of CDs were 14.6%, 29.0%, and 6.95%, respectively. Concerning the optical properties of the CDs, the UV-vis absorption and fluorescence emission and excitation spectra were recorded (Figure 1b), and revealed a maximum absorption, fluorescence excitation and emission wavelengths at 354, 368 and 462 nm, respectively. These intrinsic photoluminescence properties make it possible to follow CD fate in biological conditions without the addition of a fluorescent marker.

### 3.2. Lung Inflammation in Response to CDs

**Figure 2:**
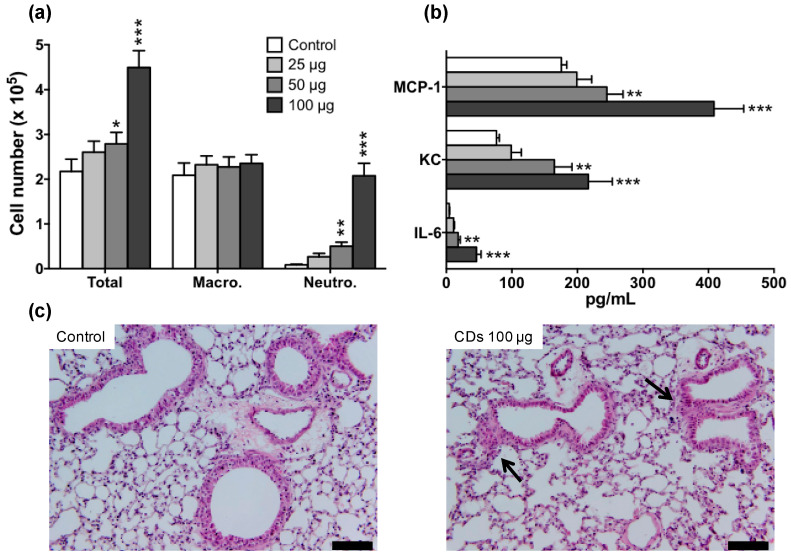
Lung inflammation induced by CDs in mice. Mice received a single administration of 25, 50 or 100 µg of CDs, and airway inflammation was assessed 24 h later. (**a**) Total and differential cell counts and (**b**) proinflammatory cytokines in BALFs. Data are means ± SEM of n = 6 mice. Statistical differences when compared to controls were determined by ANOVA followed by the Dunnett’s test. * *p* < 0.05; ** *p* < 0.01; *** *p* < 0.001. (**c**) Histopathology of lung tissues from control mice (left) or mice exposed to 100 µg CDs (right). Lung sections were stained with hematoxylin and eosin (scale bar = 50 µm; black arrow: cell infiltrate).

First, we examined whether the prepared CDs could induce an inflammatory response in the lung of mice. Animals were exposed to saline (control mice) or increasing doses of CDs (25, 50 or 100 µg), and BALFs were collected 24 h later. As shown in Figure 2a, a neutrophil infiltrate was observed in BALFs of mice that received 50 (*p* < 0.01) or 100 µg (*p* < 0.001) of CDs, when compared to the control group. In line with these findings, levels of the pro-inflammatory cytokines IL-6, KC, and MCP-1 were significantly increased in BALFs from mice exposed to 50 (*p* < 0.01) or 100 µg (*p* < 0.001) of CDs (Figure 2b). Histopathology of lung tissue from mice exposed to CDs revealed the presence of peribronchial and perivascular cellular infiltrates, which confirmed the inflammatory response found in BALFs (Figure 2c). Thus, all together, the herein described bPEI600-based CDs evoked a dose-dependent lung inflammation. This toxicological effect occurs in the same dose range as that evoked by other carbonaceous nanomaterials, namely carbon black [42,43] and carbon nanotubes [18]. Furthermore, it is in keeping with the data reported by other groups on ethylenediamine-based CDs [31], confirming that polyamine surface-passivated cationic CDs trigger adverse outcome in the lung.

### 3.3. CD Cell Target in the Airway Lumen

**Figure 3:**
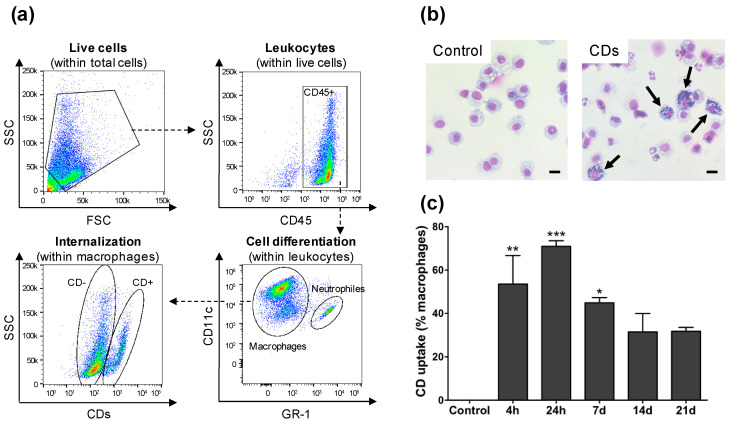
Uptake of CDs by cells of the airway lumen as studied by FACS (**a**,**c**) or optical microscopy (**b**). Mice received one administration of CDs (100 µg) or saline, and BALFs were collected 4 h, 24 h, 7 d, 14 d, or 21 d later. (**a**) Gating strategy to identify the cell types present in BALFs and to measure CD cell uptake by FACS (data from the 4-h time point). (**b**) Cells recovered from BALFs of control mice (left) or mice exposed to 100 µg CDs at the 4-h time point (right) as observed by optical microscopy. The arrows show macrophages loaded with CDs (scale bar = 10 µm). (**c**) Kinetics of internalization of CDs by alveolar macrophages as assessed by FACS. Data are means ± SEM of n = 3 mice. Statistical differences when compared to controls were determined by ANOVA followed by the Dunnett’s test. * *p* < 0.05; ** *p* < 0.01; *** *p* < 0.001.

**Figure 4:**
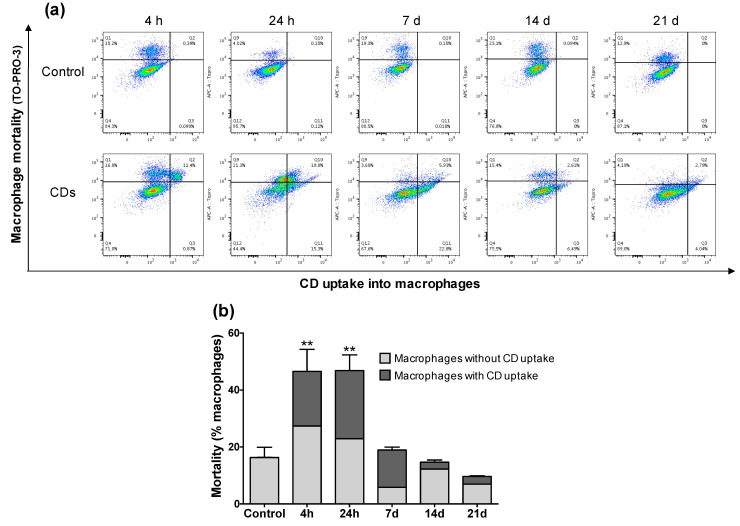
Alveolar macrophage mortality induced by CDs as a function of NP internalization as assessed by FACS. Mice received one administration of CDs (100 µg) or saline (control), and BALFs were collected 4 h, 24 h, 7 d, 14 d, or 21 d later. (**a**) CD-associated fluorescence (X-axis) as a function of TO-PRO-3 fluorescence (Y-axis) at the 4-h, 24-h, 7-d, 14-d, or 21-d time points (data from n = 1 mouse for each panel). (**b**) Kinetics of alveolar macrophage mortality in line with CD uptake. Data are means ± SEM of n = 3 mice. Statistical differences when compared to controls were determined by ANOVA followed by the Dunnett’s test, ** *p* < 0.01.

We then focused on the identification of the cells targeted by the CDs in the airways lumen of mice by analyzing cells recovered in BALFs by FACS. The staining and gating strategy made it possible to measure both the CD cell uptake (Figure 3) and mortality (Figure 4) while discriminating the different cell types. Mice received one administration of CDs (100 µg) or saline, and BALFs were collected after 4 h, 24 h, 7 d, 14 d or 21 d. As shown on Figure 3a for the 4-h time point, macrophages (CD45+CD11c+GR-1-) and neutrophils (CD45+CD11c+GR-1+) were the two main cell populations present in BALFs from mice exposed to CDs, which is in agreement with cytological data of Figure 2a. To investigate CD uptake, the CD-associated fluorescence was measured into these two cell populations. No internalization was measured within neutrophils (data not shown). In contrast, an important CD uptake was observed within macrophages (Figure 3a, panel internalization within macrophages). Examination of BALF cells from mice exposed to CDs by optical microscopy showed CD-loaded macrophages (Figure 3b), which confirmed the FACS data. Regarding the kinetics of CD internalization by macrophages, more than 50% of cells had internalize CDs as early as 4 h after instillation of the NPs in the lung of mice, and this internalization increased further at 24 h, with more than 75% of CD-loaded macrophages (Figure 3c). Presence of CDs into macrophages started to decrease from day 7 after administration, but the NPs were still present at day 21, suggesting that they are not rapidly cleared.

Macrophage mortality induced by CDs was assessed by combined analysis of CD-associated fluorescence (X-axis) and fluorescence of the TO-PRO-3 cell mortality marker (Y-axis) (Figure 4a). Thus, different macrophage populations were discriminated: living macrophages with no CD uptake; living macrophages with CD uptake; dead macrophages without CD uptake; dead macrophages with CD uptake. As shown on Figure 4b, a macrophage mortality of approximately 15% was found in control mice, which is in agreement with what is generally reported in studies performing FACS on BALFs [38]. In contrast, significant macrophage mortality was observed in BALFs from mice exposed to CDs, since cell death affected almost 50% of the total macrophage population at 4 h and 24 h after exposure to the NPs. Among the dead macrophages, between 20% and 25% of the cells contained CDs at 4 h and 24 h, respectively. These data suggest that CDs can exert a direct toxicity on macrophages after their cell uptake. However, macrophage mortality decreased sharply from 7-d post-exposure and returned to a level comparable to control at 21 d after CD exposure, suggesting that there is a possible recovery of the alveolar macrophage population from 21 d after a unique CD exposure.

Thus, we found that alveolar macrophages are the main cell target of bPEI600-based CDs in the lung. Uptake of NPs by macrophages has been reported for various kinds of nanomaterials [42,44] and is now widely accepted. As alveolar macrophages constitute the first line of defense against foreign materials in the lung [45], NP engulfing by macrophages plays certainly a central role in alveolar clearance of inhaled NPs, including carbon NPs [46,47]. However, accumulation of NPs into alveolar macrophages may also lead to a release of pro-inflammatory mediators [19]. In our study, the increased quantities of interleukins (IL-6, KC) and monocyte chemoattractant protein-1 (MCP-1) in mouse BALFs are likely due to the macrophage response to CDs, leading to a lung inflammatory process. Moreover, NPs may also cause direct damage to macrophages leading to cell death in some cases [42]. Indeed, our data show a high proportion of mortality among macrophages that had taken up CDs at 4-h and 24-h post-exposure. Although in our study macrophage mortality decreased from 7 d post CD exposure, any weakening of the alveolar macrophage population would affect pulmonary clearance thereby increasing the risk of developing lung disease [19]. In order to elucidate the mechanisms involved in macrophage toxicity, we investigated next the toxicological responses evoked by our CDs in cultured macrophages.

### 3.4. Internalization of CDs by Cultured Macrophages and Its Mechanisms

**Figure 5:**
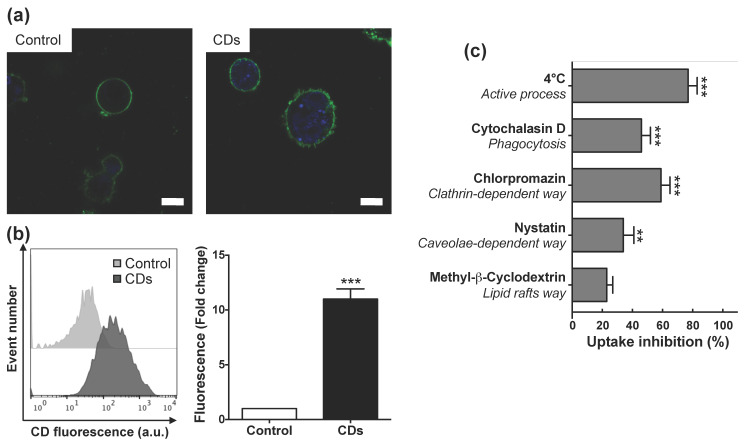
Uptake of CDs in cultured macrophages as assessed by CLSM or FACS. (**a**) CLSM analysis of unexposed cells (left) or cells exposed to 25 µg/mL of CDs for 4 h (right). Cells were stained with the fluorescent membrane probe DSQ12S before observation. Cell membrane is colored in green and CDs appear in blue (scale bar = 10 µm). (**b**) Quantification of CD internalization by FACS. Results are expressed as fold change in fluorescence intensity when compared to control cells and are means ± SEM of n = 9 experiments. Statistical differences when compared to control were determined by Student’s *t*-test. *** *p* < 0.001. (**c**) Study of CD internalization mechanisms by FACS. Cells were pre-incubated at 4 °C or pre-treated with various pharmacological inhibitors for 30 min before the addition of CDs (25 µg/mL). Internalization was measured at 4 h. Results were expressed as percent of inhibition of CD uptake and are means ± SEM of n = 3 experiments. Statistical differences when compared to control were determined by ANOVA followed by the Dunnett’s test. ** *p* < 0.01; *** *p* < 0.001.

As toxicity of NPs generally involves their cell entry [48], we first investigated cell uptake of CDs by macrophages and its associated mechanisms. Following incubation of cells with non-cytotoxic concentration (25 μg/mL) of CDs for 4 h, CDs were visible in the cytoplasm of macrophages as blue spots by CLSM (Figure 5a), whereas no such spots were visible in control cells. As shown on Figure 5b, a quantitative analysis conducted by FACS highlighted an increase in CD fluorescence signal into macrophages (10.9-fold compared to control, *p* < 0.001), which confirmed CD internalization by these cells in vitro. This uptake of CDs by cultured macrophages is in agreement with our present in vivo data, and with our previous in vitro studies reporting efficient cell uptake of bPEI25k-based CDs by macrophages [36].

The different pathways involved in CD internalization were then examined by FACS (Figure 5c). Cell incubation at 4 °C (inhibition of active process) induced a reduction in CD uptake by nearly 80%. This limited uptake of CDs at low temperature indicated an energy-dependent endocytosis of CDs. However, as this inhibition was not complete, a part of the CD uptake may be due to an energy-independent process, as for example passive diffusion. Such an effect could be explained by the very small size of our CDs (10 nm) and was previously reported for other CDs (1–5 nm) [49] or for other types of very small NPs as, e.g., gold NPs [50]. While methyl-β-cyclodextrin (inhibitor of lipid rafts) did not significantly alter CD cell uptake, cytochalasin D (inhibitor of phagocytosis), chlorpromazin (inhibitor of clathrin-mediated endocytosis) and nystatin (inhibitor of caveolae-mediated endocytosis) significantly inhibited the NP macrophage uptake by 46%, 59% and 34%, respectively (Figure 5c). This partial inhibition of CD uptake by chlorpromazin, nystatin or cytochalasin D indicates that CD internalization is the consequence of endocytosis through at least three different pathways, clathrin- and caveolae-mediated pathways, and also phagocytosis. Widely known, clathrin- and caveolin-dependent endocytosis are two major pathways for NP cellular entry [51]. If the clathrin pathway results in the engulfment of NPs in intracellular vesicles with a size around 100–500 nm, caveolae are smaller vesicles with a diameter of 50–100 nm. So, the size of NPs determines the processes of cellular uptake [52]. In the case of ultrasmall NPs such as CDs, these two endocytosis pathways can be efficient, as it was previously reported [49]. Phagocytosis is a NP uptake mechanism exercised by immune cells, including macrophages. This process results from binding of NPs to cell surface receptors of phagocytes after their opsonization by serum proteins including immunoglobulins and complement proteins [51]. Our bPEI600-based CDs exhibit a positive surface charge, which could promote their interactions with negatively charged serum proteins via electrostatic bonds, thus facilitating their opsonization and therefore their phagocytosis by macrophages [53]. Finally, our results suggest that the very small size of CDs leads to their internalization by macrophages by an energy-independent process, as well as by clathrin- and caveolin-dependent endocytosis pathways. Moreover, the cationic surface of the bPEI600-based CDs may also contribute to cellular uptake by phagocytosis.

### 3.5. Cellular Mechanisms Involved in the Toxicity of CDs towards Macrophages

**Figure 6:**
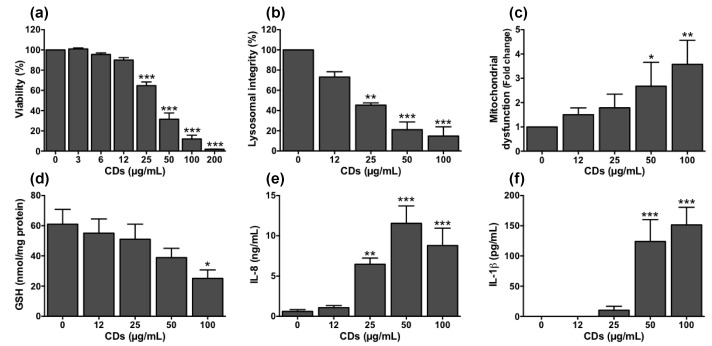
Cellular mechanisms involved in the toxicity of CDs towards cultured macrophages. Cells were exposed or not with increasing concentrations of CDs for 24 h (**a**,**b**,**e**,**f**) or 4 h (**c**,**d**). Cell viability (**a**, MTT assay), lysosomal integrity (**b**, neutral red assay), mitochondrial dysfunction (**c**, mitochondrial membrane perturbation assay), oxidative stress (**d**, reduced glutathione), inflammatory response (**e**, IL-8 secretion) and NLRP3 inflammasome activation (**f**, IL-1β secretion) were measured at 4 h (**c**,**d**) or 24 h (**a**,**b**,**e**,**f**). Data are means ± SEM of n = 3–6 experiments. They are expressed in percentage of controls (**a**,**b**), fold change (**c**), or as absolute values (**d**–**f**). Statistical differences when compared to controls were determined by ANOVA followed by the Dunnett’s test. * *p* < 0.05; ** *p* < 0.01; *** *p* < 0.001.

To evaluate the dose-dependent toxicity of CDs on cultured macrophages, cells were exposed to increasing concentrations of the NPs (3–200 µg/mL) for 24 h. These concentrations were chosen according to previous works on CD toxicity [29,33,34]. They are in line with sets of doses recommended for nanotoxicological studies conducted on submerged cell tests [54]. Furthermore, when normalized according to the nanomaterial metric doses as proposed by Loret et al. [55], they are of the same order of magnitude as the CD doses we used in our in vivo experiments. Indeed, in our experimental conditions, the in vitro CD concentration of 200 μg/mL did correspond to a CD dose of 0.4 μg/10^3^ macrophages, whereas the in vivo CD dose of 100 μg was equivalent to 0.5 μg/10^3^ macrophages taking into account a number of macrophages of 250.000 in mouse airways, as determined by cell counting in BALFs of naïve animals. As shown on Figure 6a, a decrease in cell viability of 35–85% was observed at CD concentration ranging from 25 to 100 µg/mL, and a maximum loss of viability was reached at 200 µg/mL. Thus, in agreement with our previous studies on bPEI25k-based CDs, we found that bPEI600-based CDs are toxic to macrophages [32,36]. A lactate dehydrogenase (LDH) release was measured in the cell culture supernatants of macrophages exposed to CDs (data not shown), suggesting that the cell death induced by CDs probably occurs by a necrosis mechanism. In this type of CDs, the presence of polyamine groups at the CD surface results in a highly cationic surface of the particles, which may promote the interactions of CDs with the negatively charged cellular membranes and then their internalization, as discussed above. In addition, it was found that a positive surface charge could promote their interactions with negatively charged serum proteins via electrostatic bonds and may facilize their opsonization and therefore their phagocytosis by macrophages [53]. In the literature, other works supported this charge effect hypothesis. Indeed, Havrdova et al. showed that poly(ethyleneimine)-based CDs exhibiting positive surface charge induced greater toxicity as compared to negative or neutral ones [29]. A recent toxicogenomics study conducted in human lung fibroblasts revealed also that positive CDs induced changes of immune response and cell cycle-related processes, contrary to negative ones [56].

Next, we investigated the mechanisms leading to these viability losses using CD concentrations ranging from those causing little viability loss (12 µg/mL) to submaximal cytotoxic (100 µg/mL) concentrations. As the lysosome and the mitochondria are the main subcellar targets of NPs [57], we examined whether the decrease in cell viability induced by our CDs was accompanied by some disturbance of these two organelles. A significant loss in lysosomal integrity (Figure 6b) and an increase in mitochondrial dysfunction (Figure 6c) were observed in response to CDs. These effects were dose-dependent and significant from the CD concentration of 25 µg/mL and 50 µg/mL for lysosomal and mitochondrial perturbation, respectively. Induction of oxidative stress is another major mechanism of cell death evoked by NPs [20]. In order to assess the ability of CDs to induce this response, the cellular content in reduced glutathione (GSH), an anti-oxidant agent, was quantified. As shown on Figure 6d, CDs induced a dose-dependent decrease in GSH cell content reaching almost 40% at 100 μg/mL, indicating the occurrence of oxidative stress. Next, to investigate the inflammatory effect of CDs, the levels of IL-8, which is one of the main macrophage-derived inflammatory cytokines, were measured in the supernatants of CD-exposed macrophages. As shown on Figure 6e, macrophage exposure to CDs resulted in a dose-dependent increase in IL-8 secretion with a significant effect from the concentration of 25 µg/mL. Finally, the impact of CDs on NLRP3 inflammasome was evaluated by quantifying the IL-1β cytokine in the culture supernatants of macrophages. Indeed, NLRP3 inflammasome is a cytosolic multiprotein complex, which has a key role in the regulation of systemic and local responses to infection and injury. As illustrated on Figure 6f, IL-1 β was secreted by macrophages in response to CDs (50–100 µg/mL), highlighting that CDs can induce NLRP3 inflammasome activation.

We thus identified several adverse cellular effects of bPEI600-based CDs in macrophages. The first major organelle KE in response to CDs was disruption of the lysosome integrity. It is well known that the lysosomal membrane presents a high sensitivity to xenobiotics. Hence, lysosomal membrane integrity has already been proposed as a biomarker to evaluate potential effect of the environmental pollutants [58]. Recently, the role of lysosomal dysfunction in NP toxicity has been underlined in the literature [59]. Indeed, since endocytosis is the main way for NPs to enter cells, and since lysosomes are the terminal components of the endo/lysosomal system, most NPs end-up and accumulate in lysosomes after their cell uptake [60]. Although the exact mechanisms leading to lysosome damage are not fully understood, this trafficking to the lysosomes, associated with loss of lysosomal integrity, has been reported for NPs of various chemical composition including carbon-based NPs [61,62]. These data are also consistent with our previous work indicating that bPEI25k-based CDs were addressed to the lysosomes after their uptake by macrophages and caused a loss of lysosomal integrity [36].

A second major organelle KE induced by bPEI600-based CDs in the present study is mitochondrial dysfunction. Mitochondrial dysfunction is reported as a crucial mechanism leading to toxicity of NPs [57], including carbon-based NPs [42]. In line with our data, a recent study reported that N-doped CDs caused a decline in mitochondrial activity in hepatocytes [33]. Mitochondrial damage in response to CDs could be a direct effect of these NPs on the organelle, as a silica nanoplatform conjugated to fluorescent CDs has been observed to accumulate in mitochondria in several human cell lines [63]. It is also possible that CDs affect the mitochondria by indirect pathways. Supporting this hypothesis, we previously reported that inhibition of the lysosomal protease cathepsin B significantly reduced mitochondrial dysfunction induced by bPEI25k-based CDs [36].

Another cellular KE we observed in macrophages exposed to bPEI600-based CDs is oxidative stress. Oxidative stress is undoubtedly one of most frequently reported adverse effect among all toxicity endpoints studied in the nanotoxicology literature [20,64]. Its involvement has already been mentioned in toxicity studies conducted on N-doped CDs obtained from carbon nitride [33] or poly(ethyleneimine)-based CDs [29,36]. It has been reported that NP-induced oxidative stress may result from the production of reactive species such as ROS and/or reactive nitrogen species (RNS), or from the alteration of antioxidant activities within the cell [20,48]. In our study, the mitochondria dysfunction induced by CDs could result in excessive production of ROS through electron carriers from the electron transfer chain, as it has been recently reported for other types of NPs [65]. The CD-induced oxidative stress may also result from the damage to other organelles, in particular the lysosome, as described for other NPs [48].

In the present study, among the mechanisms incriminated in macrophage toxicity of bPEI600-based CDs, NLRP3 inflammasome activation also appeared as a KE, as it could participate to the inflammatory response evoked by the NPs. These last years, several kinds of NPs have been reported to activate the NLRP3 inflammasome, and nanoparticle-associated molecular patterns (NAMPs) have been proposed to be new danger signals leading to NLRP3 inflammasome activation [66]. Among possible NLRP3 activation mechanisms, ROS production [67] or destabilization of lysosomes and release of cathepsin B into macrophage cytoplasm have been proposed in the case of carbon-based nanomaterials [68], including CDs [36].

## 4. Conclusions

**Figure 7:**
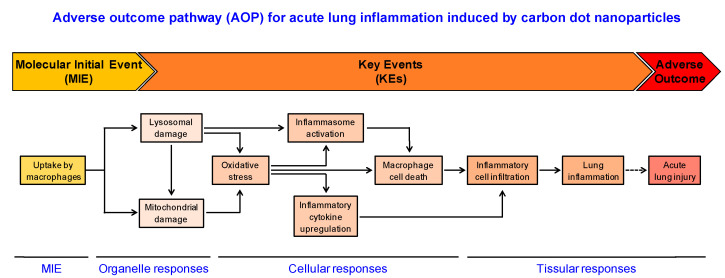
Putative AOP related to the acute lung inflammation induced by polyamine-based cationic CDs. The uptake of CDs by macrophages constitutes the MIE, followed by the KEs, which are the loss of lysosomal integrity and mitochondrial disruption (organelle responses), oxidative stress, inflammasome activation, inflammatory cytokine upregulation and macrophage death (cellular responses). All these effects may trigger inflammatory cell infiltration, leading to lung inflammation (tissular responses).

In this study, the combination of in vivo and in vitro experimental approaches was used to propose an AOP related to the acute lung injury induced by polyamine-based cationic CDs (Figure 7). Based on our data, we propose that uptake of CDs by macrophages constitutes the MIE of this pathway, followed by the loss of lysosomal integrity, mitochondrial disruption, oxidative stress, and inflammasome activation as KEs. All these effects may trigger macrophage death, leading to lung inflammation. This putative AOP is aligned with the recommended KEs that emerged from the network of inflammation-related AOPs [69]. Besides, as lung inflammation may lead to acute lung injury, lung fibrosis or lung cancer, it could be extended and/or connected to other larger networks in the field of inhalation toxicology in the AOP-Wiki database. Although each NP exhibits specific physicochemical properties, such as size and surface charge or chemistry that govern their interactions with biological environment and influence their toxicity potential [70], the AOP we propose could be useful for providing clarity on lung toxicity mechanisms for other types of NPs than CDs, especially other cationic NPs. In addition, some KEs described in this study such as oxidative stress or inflammation are described in AOPs under development for lung toxicity induced by chemicals [71]. But our data highlight some major differences between the toxicodynamics of chemical compounds and NPs. These differences lie in particular within the MIE (cell uptake of NPs) and the early KE (lysosomal dysfunction induced by NPs). Thus, in our opinion, the integration of specific KEs related to nanomaterials inside pre-existing AOPs, and also the development of AOPs specific to nanomaterials should be encouraged in order to provide new insights to gain a better understanding of NP toxicity. This is valuable for identifying endpoints and targeted biomarkers for nanosafety assessment.

## Figures and Tables

**Table 1:** Physicochemical characteristics of CDs.

Characteristics	Data
Size	DLS: 10.1 ± 0.4 nm, PdI = 0.240 ± 0.061 TEM: 39.5 nm
Surface charge	Zeta potential: ζ = +23.9 ± 2.0 mV
Chemical composition	Nitrogen: 14.6% Carbon: 29.0% Hydrogen: 6.95%
Photoluminescence	λ_max_: 354 nm λ_ex_: 368 nm λ_em_: 462 nm

## Data Availability

Data presented in this study are available by requesting from the corresponding author.
